# Expression of Human Placenta-specific 1 (PLAC1) in CHO-K1 Cells

**Published:** 2020

**Authors:** Jafar Mahmoudian, Mahboobeh Nazari, Roya Ghods, Mahmood Jeddi-Tehrani, Seyed Nasser Ostad, Mohammad Hossein Ghahremani, Sedigheh Vafaei, Mohammad Mehdi Amiri, Amir-Hassan Zarnani

**Affiliations:** 1. Nanotechnology Research Center, Faculty of Pharmacy, Tehran University of Medical Sciences (TUMS), Tehran, Iran; 2. Monoclonal Antibody Research Center, Avicenna Research Institute (ACECR), Tehran, Iran; 3. Oncopathology Research Center, Iran University of Medical Sciences (IUMS), Tehran, Iran; 4. Department of Molecular Medicine, Faculty of Advanced Technologies in Medicine, Iran University of Medical Sciences (IUMS), Tehran, Iran; 5. Department of Toxicology and Pharmacology, Faculty of Pharmacy, Tehran University of Medical Sciences, Tehran, Iran; 6. Reproductive Immunology Research Center, Avicenna Research Institute, (ACECR), Tehran, Iran; 7. Department of Immunology, School of Public Health, Tehran University of Medical Sciences (TUMS), Tehran, Iran; 8. Immunology Research Center (IRC), Iran University of Medical Sciences (IUMS), Tehran, Iran

**Keywords:** Eukaryotic cells, PLAC1 protein expression, Protein transport

## Abstract

**Background:**

Placenta-specific 1 (PLAC1), as a new Cancer/Testis Antigen (CTA), is frequently expressed in a variety of cancers and localized to cytoplasm and plasma membrane. Surface expression of cancer target antigens is of great importance that enables antibody-mediated cancer immunotherapy. The aim of the current study was to express the intact human PLAC1 protein on plasma membrane of a eukaryotic cell as a model for future anti-PLAC1-based cancer immunotherapy.

**Methods:**

In the first approach, entire human PLAC1 gene including its own Signal Peptide (SP) was cloned into pIRES2-EGFP and LeGO-iG2 vectors and expressed in CHO-K1 cells. In the second approach, cytosolic and Signal-Anchor (SA) sequence of Transferrin Receptor Protein 1 (TFR1) were fused to extracellular portion of PLAC1 and expressed as above. Expression of PLAC1 was then assessed using Reverse Transcription Polymerase Chain Reaction (RT-PCR), Western Blot (WB), Immunocytochemistry (ICC), Immunofluorescence (IF) and Flow Cytometry (FC).

**Results:**

The first approach resulted in the expression of PLAC1 in submembranous but not in the surface of transfected CHO-K1 cells. Using the chimeric human PLAC1 construct, the same intracellular expression pattern was observed.

**Conclusion:**

These results indicated that there are some yet unknown PLAC1 localization signals employed by cancer cells for surface expression of PLAC1.

## Introduction

Surgery and chemotherapy are among well-established therapeutic modalities for most of the patients with cancer and considerably have increased survival rate of cancer patients. However, all these therapeutic approaches are associated with various undesirable side effects on normal tissues. In this regard, the exploiting of the immune system to specifically eradicate cancer cells remains an interesting option. Using tumor antigens is one of the most exciting approaches to overcome cancer by anti-tumor T cell responses. Tumor antigens may originate from mutated, overexpressed or aberrantly expressed normal proteins 1,2. Tumor-Associated Antigens (TAAs) 3, tumor-specific antigens (TSAs) 1,3, and Cancer/Testis Antigens (CTAs) 4,5 are the main classes of tumor antigens. The lack of bonafide TSAs, however, is a main obstacle in cancer immuno therapy. CTAs are expressed in gametes and tropholasts and also in many types of cancers 2. Notably, CTAs have captured the focus of many researchers during the past few years with encouraging results 5,6. In the scarcity of tumor-specific neoantigens, the over-expression of CTAs by cancer cells to trigger an anti-tumor immune response remains an encouraging case for the researchers 7.

Placenta-specific 1 (PLAC1) is a new member of cancer testis antigens which was first introduced by Cocchia *et al* in 2000 8. Human PLAC1 maps 65 *kb* telomeric to hypoxanthine-guanine phosphoribosyl transferase (HGPRT) at Xq26 and encodes a small protein consisting of 212 amino acids 8. PLAC1 protein is mainly expressed in placenta 9–12, while it is frequently activated in a variety of cancers including cancers of breast 13–16, lung 13–15,17,18, liver 14,17,19, colon 14,15,17,20–22, stomach 13,23,24, ovary 13,25,26, uterus 27,28, cervix 14,29, pancreas 30, and prostate 31,32. PLAC1 is an important oncogenic factor and its expression is associated with invasiveness, metastasis, and proliferation of cancer cells 13,19 and is positively correlated with clinic-pathological parameters of some cancer types 18,24, 31,33.

Various PLAC1 protein localizations have been reported in cancer cells and tissues including nucleus 22,24, cytoplasm 19,20,24,30,34, and plasma membrane 13,14, 34,35. Surface expression of cancer target antigens is of great importance that enables antibody-mediated cancer immunotherapy. The aim of the present study was to express the intact human PLAC1 protein on plasma membrane of a eukaryotic cell as a model for future anti-PLAC1-based cancer immunotherapy.

## Materials and Methods

### Cell lines and culture conditions

CHO-K1, MCF7, and MDA-MB-231 cell lines were provided by the National Cell Bank of Iran (Pasteur Institute of Iran, Tehran, Iran). CHO-K1 and MCF7 cell lines were cultured in RPMI 1640 (Gibco, Invitrogen, CA, USA) and MDA-MB-231 cells in DMEM-F12 (Gibco) media. All media were supplemented with 10% Fetal Bovine Serum (FBS) (Gibco), 100 *U/mL* penicillin, and 100 *μg/ml* streptomycin in a humidified incubator at 37°*C* with 5% CO_2_.

### Construction of expression vectors for full human PLAC1 protein

RNA was extracted from MCF7cells using ambion PureLink RNA Mini Kit (Thermo Fisher Scientific, Waltham, MA, USA) according to the manufacturer’s recommendations. RNA integrity was confirmed by agarose gel electrophoresis and the concentration was determined by measuring the Optical Density (OD) at 260 *nm* in a NanoDrop spectrophotometer (Thermo Fisher Scientific). First strand cDNA was synthesized using ∼3 *μg* (10 *μl*) of RNA, 4 *μl* 5X reaction buffer (Thermo Fisher Scientific), 2 *μl* dNTPs (Roche, Basel, Switzerland), 1 *μl* N6 random hexamers (Thermo Fisher Scientific), 1 *μl* reverse transcriptase (Thermo Fisher Scientific), and 2 *μl* water in a total volume of 20 *μl* as follows: 10 *min* at 25*°C*, 60 *min* at 42*°C* and 10 *min* at 70*°C*. The sequence of primers for amplification of PLAC1 was as follows: sense5′-ATATGCTAGCGC CACCATGGGCATGAAAGTTTTTAAGTTTATAA CTGATG-3′ (with NheI restriction site, Kozak sequence, and start codon) and antisense 5′-TATGGA TCCTCAGTGGTGGTGGTGGTGGTGCATGGACC CAATCATATCATC-3′ (with BamH1 restriction site, stop codon, and His-tag sequence). The PCR amplification was carried out under the following conditions: initial denaturation at 94*°C* for 5 *min*, a 30- cycle amplification (98*°C* for 10 *s*, 55*°C* for 30 *s*, and 72*°C* for 30 *s*), and a final extension for 5 *min* at 72*°C*. PCR reactions were performed in a 20 *μl* volume containing 1 *μl* cDNA, 0.25 *μl* (10 *pmoles/μl*) of each primer, 8.5 *μl* water, and 10 *μl* Taq DNA Polymerase Master Mix RED (Ampliqon, Odense, Denmark). Amplicons were digested byNheI/BamH1 and inserted into the digested/dephosphorylated pIRES2-EGFP (Takara Bio, Mountain View, CA, USA) andLeGO-iG2 (Addgene, Cambridge, MA, USA) expression vectors. The ligated mixtures were chemically transformed into *E. coli* DH5 alpha. Positive colonies were screened using colony PCR experiment. Finally, plasmids were extracted and further confirmed through double digestion and sequencing experiments.

In the second approach, the pIRES2-EGFP vector was engineered to display the chimeric PLAC1 protein on the plasma membrane of CHO-K1 cells. Chimeric PLAC1 (TR-PLAC1) composed of cytoplasmic and SA sequence of TFR1 (aa 1–99) was fused in-frame to truncated PLAC1 protein (aa 50–212). The TR-PLAC1 sequence was codon-optimized and cloned into pIRE-S2-EGFP vector by Biomatik Company (Ontario, Canada) where the construct was finally confirmed using double digestion and sequencing analysis.

### Transient transfection and generation of stable cell line

CHO-K1 cells were transfected with pIRES2-EGFP-TR-PLAC1, pIRES2-EGFP-PLAC1, LeGO-iG2-PLAC1 or respective empty vectors using lipofectamine 2000 or 3000 (Thermo Fisher Scientific) according to the manufacturer’s protocol. After 24 or 48 *hr*, transiently transfected cells were used for RT-PCR, WB, ICC, IF, and/or flow cytometric analysis. For polyclonal stable cell line generation, transiently transfected cells were treated with 900 *μg/ml* G418 (Sigma, St. Louis, MA, USA) for14 days.

### Reverse transcription polymerase chain reaction (RT-PCR)

CHO-K1 cells were transiently transfected using pIRES2-EGFP-PLAC1, LeGO-iG2-PLAC1 or respective empty vectors in 12-well plates. Twenty-four *hr* after transfection, cells were harvested using trypsine-EDTA and RNA was extracted as described above. DNA contamination was removed using a commercial kit (Sigma, Product Number: AMPD1) according to the manufacturer′s recommendation. cDNA was synthesized as described above and then used for PLAC1 amplification with the following primers: sense 5′-ATTACATATGGCCCCCCAAAAGTCCCCATG-3′ and antisense 5′-ATAAAGCTTTCACATGGACCCA ATCATATCATC-3′. The following PCR program was used for DNA amplification: 94*°C* for 5 *min*; 30 cycles at 98*°C* for 10 *s*, 60*°C* for 30 *s*, 72*°C* for 30 *s*; and a final extension at 72*°C* for 2 *min*. PCR reactions were performed in a 20 *μl* volume containing 2 *μl* cDNA, 0.5 *μl* (10 *pmoles/μl*) of each primer, 7 *μl* water, and 10 *μl* Taq DNA Polymerase Master Mix RED (Ampliqon).

As an internal control, β-actin was amplified. A 366 *bp* β-actin PCR product was amplified using sense 5′-GCAAGAGATGGCCACTGCCGC-3′and antisense 5′-GCTGACAGGATG-CAGAAGGAGA-3′ primers. The PCR amplification was carried out using initial denaturation at 94*°C* for 5 *min*, a 25- cycle amplification (98*°C* for 10 *s*, 60*°C* for 20 *s*, and 72*°C* for 25 *s*), and a final extension for 5 *min* at 72*°C*. PCR reactions were performed in a 20 *μl* volume containing 2 *μl* cDNA, 0.25 *μl* (10 *pmoles*/*μl*) of each primer, 7.5 *μl* water, and 10 *μl* Taq DNA Polymerase Master Mix RED (Ampliqon). PCR products (expected product size 291 *bp* for PLAC1 and 366 *bp* for β-actin) were evaluated using 1% agarose gel electrophoresis.

### Western blot

Western blot analysis was done as described elsewhere 36–38. Briefly, transfected cells were harvested twenty-four *hr* after transfection with saline sodium citrate buffer pH=8.0 (15 *mM* sodium citrate and 130 *mM* potassium chloride) and washed three times with cold PBS. 1×106 cells were lysed in 50 *μl* sample buffer. Twenty *μl* of cell lysates and 28 *ng* recombinant human PLAC1 (rhPLAC1) 36 were run on a 15% SDS-PAGE gel. The membrane was then probed with either rabbit anti-rhPLAC1 antibody 32 (2 *μg*/*ml* for 1.5 *hrs*) followed by goat anti-rabbit IgG-HRP (1:3000) (Bio-Rad, Hercules, CA, USA) or with HRP-conjugated anti-his tag antibody (HRP anti-his tag Ab) (Sina Biotech, Tehran, Iran) at 1/2000 dilution for 1.5 *hr*. Anti-β-actin (clone: D6A8) rabbit monoclonal antibody (1:2000) (Cell Signaling Technology, Denver, MA, USA) and goat anti-rabbit IgG-HRP (1:3000) (Bio-Rad) were used for visualization of β-actin. Signals were developed by Immobilon Western Chemiluminescent HRP Substrate detection system (Merck millipore, Burlington, Massachusetts, USA) according to the manufacturer’s instruction.

### R-phycoerythrin (R-PE)-anti-rhPLAC1 Ab conjugation

R-PE protein (Thermo Fisher Scientific) was conjugated to anti-rhPLAC1 Ab 39,40. In brief, five hundred micro-litter R-PE (2 *mg*/*ml*) was mixed with 200 *μg* sulfo-MBS bifunctional crosslinker (Thermo Fisher Scientific) for 2 *hr* followed by dialysis against PBS/EDTA *5 mM*. The antibody was partially reduced using DTT and then mixed with activated R-PE at 1:2 molar ratio (1 *mole* antibody and 2 *moles* activated R-PE) for 2 *hr*. Conjugated antibody was extensively dialyzed against PBS/EDTA 5 *mM*.

### Immunocytochemistry (ICC) and immunofluorescence (IF)

ICC and IF were done as described elsewhere 41–43. Twenty-four *hr* after transfection, cells were grown on a slide and fixed using formaldehyde. In ICC staining, slides were blocked with 5% normal mouse serum for 1 *hr* and then incubated with HRP anti-his tag Ab (1:200) (Sina Biotech) for 2 *hr*. After washing, signals were developed by adding Diaminobenzidine (DAB). Digital images were captured by IX71 microscope (Olympus, Tokyo, Japan). In IF staining, cells were fixed as above, incubated with 10 *μg/ml* anti-rhPLAC1 Ab for 1 *hr* followed by addition of PE-labeled goat anti-rabbit Ig (1:100) (Razi Biotech, Tehran, Iran). Microscopy images were acquired by Olympus IX71 microscope (Olympus).

### Flow cytometry

Briefly, cells were harvested with saline sodium citrate buffer pH 8.0 and incubated with 5% sheep serum for 30 *min* 42,44. Cells were subsequently incubatedwithPE-anti-rhPLAC1 Ab (5 *μg/ml*), PE-anti-his tag antibody (Biolegend, San Diego, CA, USA) (dilution: 1:100) or PE-isotype control (5 *μg/ml*) (Sina Biotech) for one *hr*. For intracellular PLAC1 staining, cells were fixed using 1.5% formaldehyde for 15 *min*. Cells were then permeabilized using 0.5% saponin for 15 *min*, incubated with 5% sheep serum for 30 *min* followed by PE-anti-rhPLAC1 Ab (5 *μg/ml*) incubation for one hr. Cells were analyzed by a flow-cytometer (Partec, Munster, Germany).

## Results

### Constructs containing human PLAC1 signal peptide yielded stably transfected CHO-K1 cells

**Figure 1.**
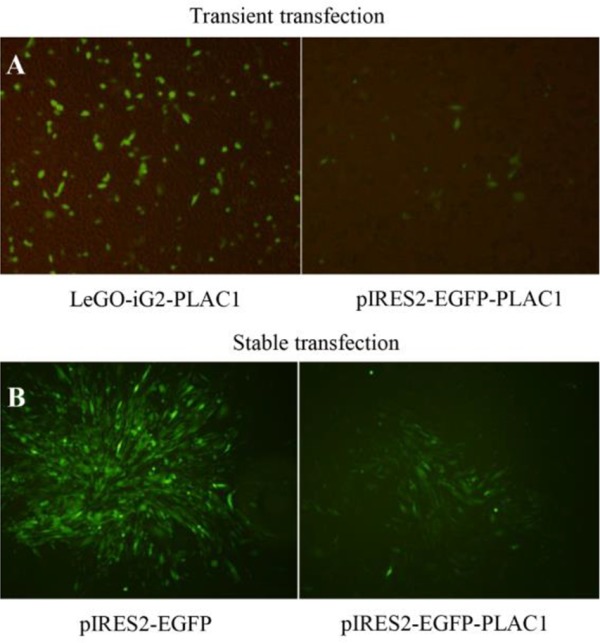
Transient and stable transfection of CHO-K1 cells with human PLAC1. (A) Twenty-four *hr* post-transfection, CHO-K1 cells showed higher frequency of transfected cells using LeGO-iG2-PLAC1 than in pIRES2-EGFP-PLAC1. (B) pIRES2-EGFP- and pIRES2-EGFP-PLAC1- transfected CHO-K1 cells were treated with G418 antibiotic to produce polyclonal stable cell line.

CHO-K1 cells were transfected using pIRES2-EGFP-PLAC1, LeGO-iG2-PLAC1, or their respective empty vectors. Our results showed that there was no difference in the EGFP signal of transfected cells when examined either 24 or 48-*hr* post-transfection (data not shown). In this experiment, 24 *hr* post-transfection CHO-K1 cells displayed more transfected cells in LeGO-iG2-PLAC1 than in pIRES2-EGFP-PLAC1 (Figure 1A). 24 *hr* post-transfection cells were used for RT-PCR, WB, ICC, IF, and FC experiments. pIRES2-EGFP- and pIRES2-EGFP-PLAC1- transfected CHO-K1 cells were further treated with G418 to produce stable cells (Figure 1B).

### PLAC1 was expressed in submembranous expression but not in the surface of transfected CHO-K1 cells

**Figure 2.**
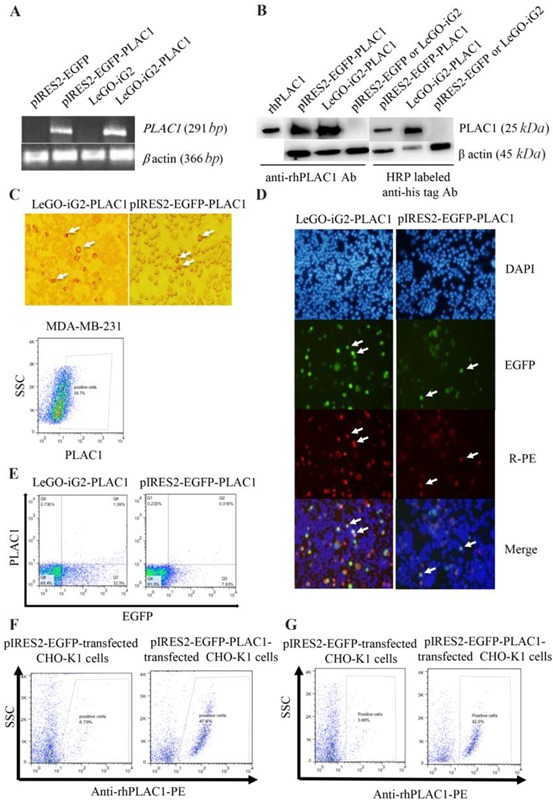
Expression and localization of full human PLAC1 in pIRES2-EGFP-PLAC1- and LeGO-iG2-PLAC1- transfected CHO-K1 cells. (A) The presence of human *PLAC1* transcript was showed by RT-PCR. (B) PLAC1 protein expression was confirmed by two different antibodies by WB. (C) Submembranous PLAC1 protein expression indicating by ICC. Arrows show the positive cells. (D) The expression of PLAC1 protein was also showed using IF. DAPI stains cell nucleus. Transiently transfected cells indicated by EGFP expression were stained using anti-rhPLAC1 Ab. (E) Cell surface flow cytometry staining revealed that PLAC1 protein was not localized on plasma membrane but intracellular flow cytometry staining confirmed cytoplasmic localization of the expressed PLAC1 protein in successive passages (F and G).

Using RT-PCR, the presence of human PLAC1 transcript in pIRES2-EGFP-PLAC1 and LeGO-iG2-PLAC1 transfected CHO-K1 cells was tested. Our data clearly revealed that human PLAC1 transcript was expressed in CHO-K1 cells transfected with both vectors as opposed to their respective empty vectors. *B actin* gene was used as a housekeeping internal control (Figure 2A). After confirming the expression of human PLAC1 transcript, the expression of PLAC1 at the protein level was separately investigated by two different antibodies by WB. Both PLAC1 transfected cells showed the PLAC1 protein band of about 25 *kDa* when probed with anti-rhPLAC1 Ab or HRP labeled anti-his tag antibody (Figure 2B). In this experiment, rhPLAC1 protein was used as a positive control for anti-rhPLAC1 Ab. The expression of PLAC1 protein was further confirmed by ICC and IF. In ICC experiment, since the produced human PLAC1 proteins entailed his-tag, HRP-labeled anti-his tag Ab was directly used to localize PLAC1 protein. Both pIRES2-EGFP-PLA-C1- and LeGO-iG2-PLAC1- transfected CHO-K1 cells showed submembranous expression of human PLAC1 protein as shown in figure 2C. Immunofluorescent experiment clearly showed that both pIRES2-EGFP-PLAC1- and LeGO-iG2-PLAC1- transfected CHO-K1 cells expressed human PLAC1 protein (Figure 2D). Cell surface and intracellular flow cytometry staining were employed to assess human PLAC1 localization in transfected CHO-K1 cells. The results of cell surface staining showed that the human PLAC1 protein was not localized on the cell membrane of transfected CHO-K1 cells (Figure 2E); in contrast, intracellular staining revealed cytoplasmic localization of the expressed PLAC1 protein (Figure 2F). MDA-MB-231 cells were used as a positive control. In order to confirm the stable expression of PLAC1in stably-transfected cells, the cells were passaged for 2 generations and PLAC1expression was monitored by flow cytometry. The results showed that successive passage of PLAC1-transfected cells do not lead to the obvious changes in PLAC1 expression (Figure 2G).

### TR-PLAC1 chimeric protein was not expressed in cell surface

**Figure 3.**
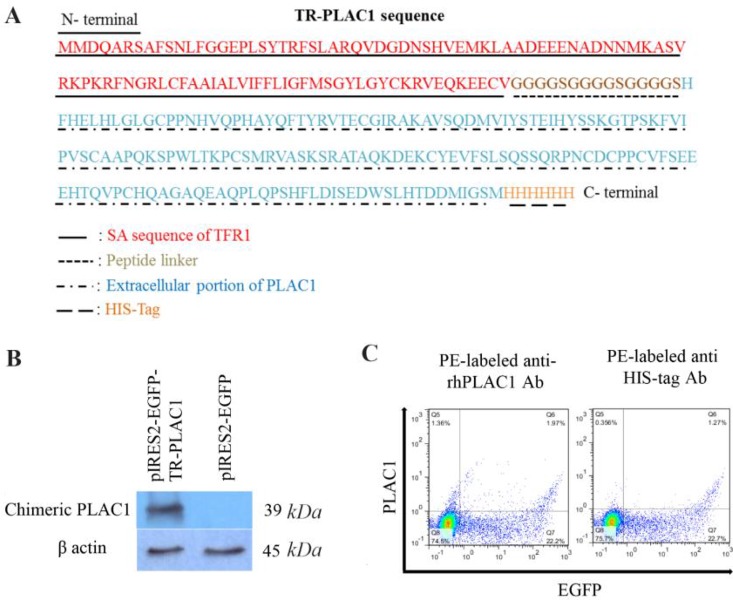
Localization of TR-PLAC1 chimeric protein. (A) Amino acid sequence of TR-PLAC1 consisting of a portion of transferrin receptor protein 1 (aa 1–99 shown in red), peptide linker ((G4S)3 shown in black), extracellular portion of PLAC1 (aa 50–212 shown in green), and 6His-Tag (shown in blue). (B) WB analysis showing expression of TR-PLAC1 protein (39 *kDa*). β-actin was used as an internal control. (C) CHO-K1 cells were transfected by lipofectamin 3000 and cell surface expression of TR-PLAC1 was examined using two different PE-labeled antibodies

As shown in figure 3A, the full-length TR-PLAC1 protein composed of cytosolic and SA sequence of TFR1 fused to extracellular portion of PLAC1 followed by six histidine (6H) amino acids at the C terminal of the protein. This construct was transfected to CHO-K1 cells using lipofectamine 3000. The expression of TR-PLAC1 was examined in CHO-K1 cells using WB (Figure 3B). The Molecular Weight (MW) of the expressed TR-PLAC1 protein (39 *kDa*) was slightly higher than its predicted MW (31 *kDa*) using ProtParam tool (ExPASy), which may be interpreted by post-translational modification during eukaryotic expression. Confirmation of TR-PLAC1 protein expression in WB was followed by flow cytometry localization of PLAC1 in transfected CHO-K1 cells. Although TR-PLAC1 was engineered to express the chimeric protein on cell surface, flow cytometric analysis showed that the chimeric protein did not localize on the surface of the CHO-K1 cell (Figure 3C).

## Discussion

The preliminary aim of this study was to express the intact human PLAC1 protein on the surface of cancer cells as a model for future anti-PLAC1-based cancer immunotherapy. To this end, intact human PLAC1 encompassing its own signal peptide was successfully cloned in two different expression vectors and the PLAC1 protein expression was confirmed in transfected CHO-K1 cells. Our data showed that despite the expression of PLAC1 in transfected cells, this protein was not localized on the plasma membrane. Regarding PLAC1 protein, as a type II membrane protein 13. In the next attempt, therefore, cytoplasmic and SA sequence of TFR1, which is a type II membrane protein, 45 was fused to extracellular portion of PLAC1 in order to express human PLAC1 on the plasma membrane. Again, despite the expression of fusion PLAC1 protein, FC data showed that this chimeric protein was not localized on the plasma membrane.

Human PLAC1 protein at subcellular level was reported to be localized in cell nucleus, cytoplasm, and plasma membrane in normal and cancer tissues. Using western blot analysis in placental tissue, PLAC1 was found to be localized in the microsomal fraction suggesting membranous localization of PLAC1 protein 35. In parallel with previous data, our team and other investigators, using polyclonal antibodies raised against amino acids 125–212 14 and 166-177 of PLAC1 protein 34, showed that PLAC1 was expressed in cell surface of syncytiotrophoblasts and cytotrophoblasts. Plasma membrane localization of PLAC1 has also been reported using siRNA silencing in breast cancer cell lines, MCF-7 and BT-549 13. It has previously been shown that PLAC1 protein was localized to cell surface of about 30% of prostate cancer cells, LNCaP, DU145 and PC3 cell lines 32 and plasma membrane in ovarian cancer cells, Caov-4 34. Using Immunohistochemistry (IHC), cell surface localization of PLAC1 protein has also been reported in prostate 31, breast 13, liver 19, and colon 20 tumors, although exact localization by IHC staining is not reliable due to the technical limitations. In contrast, there are plenty of reports indicating PLAC1 localization into cytoplasmic compartments of cancer tissues and cells 19,20,24,30,34. PLAC1 nuclear localization has also been reported in stomach adenocarcinoma 24 and in colorectal adenocarcinoma 22 tissues.

The reason for heterogeneous localization of PLA-C1 in different cells remains unclear. Protein distribution among cellular compartments depends on some factors including: 1) type and number of localization signals on the protein, 2) the relative strength of each signal, 3) the concentration of freely diffusing molecules, 4) and the concentration and activity of localization signal receptors 46. Up to now, different molecular weighs for PLAC1protein have been reported including 24 *kDa* 34, 25.6 *kDa* 20, 26 *kDa* 13, 27 *kDa* 32, and 28–30 *kDa* 35. It is unclear that various PLAC1 molecular weights reflect its different isoforms or different types of Post-Translational Modifications (PTMs). There are reports 47–49 showing that aberrant protein glycosylation in malignant cells is associated with changes in protein sorting and trafficking 50. Different isoforms of a given protein may also have different localizations 51,52. It is therefore hypothesized that different PLAC1 isoforms or PTM may lead to different localizations. Additionally, there are plenty of reports indicating that some proteins are co-localized with other proteins because of their involvement in common functional pathways 53–55. It is not clear whether artificial transfection of PLAC1 in CHO-K1 cells could establish potential interaction with other proteins leading to surface expression. Furthermore, investigators showed that proteins cleaved by proteases and subsequently the smaller polypeptides were localized in different cell compartments 54. Whether such mechanism is the cause for cytoplasmic expression of PLAC1in transfected cells needs further investigations.

## Conclusion

Taken together, it seems that localization of PLAC1 may be a function of different variables including PTM, different isoforms, co-localization with other proteins and proteolysis. Different localizations of the same protein in different cells may reflect different functions 53. In this regard, it can be deduced that artificial transfection may not necessarily lead to the expression of a protein with the same function as with original protein.
